# Evaluation of the Performance of Novel Gram-Negative and Gram-Positive Sepsis Panels for the Rapid Diagnosis of Bloodstream Infections

**DOI:** 10.3390/diagnostics16030481

**Published:** 2026-02-05

**Authors:** Chiara Chilleri, Sara Salvetti, Marco Coppi, Iolanda Montenora, Tommaso Giani, Gian Maria Rossolini, Alberto Antonelli

**Affiliations:** 1 Department of Experimental and Clinical Medicine, University of Florence, 50134 Florence, Italy; chiara.chilleri@unifi.it (C.C.); marco.coppi@unifi.it (M.C.); tommaso.giani@unifi.it (T.G.); gianmaria.rossolini@unifi.it (G.M.R.); 2 Microbiology and Virology Unit, Careggi University Hospital, 50134 Florence, Italy; 3 Microbiology Unit, San Giuseppe Hospital, 50053 Empoli, Italy; sara.salvetti@unifi.it (S.S.); iolanda.montenora@uslcentro.toscana.it (I.M.)

**Keywords:** syndromic panel, positive blood culture, species identification, resistance mechanism detection

## Abstract

**Background/Objectives:** Bloodstream infections (BSIs) are a global healthcare issue associated with high mortality rates. Rapid diagnosis is of importance for the early selection of targeted therapy to improve patient outcomes. The use of rapid molecular assays with positive blood culture (BC) allows the identification (ID) of pathogens and the most relevant resistance determinants (RDs) in a shorter turnaround time, compared to standard culture. In this study, the performances of a new syndromic panel to determine the IDs and RDs of Gram-negative (GN) and Gram-positive (GP) bacteria were investigated in comparison with a standard-of-care (SoC) workflow. **Methods:** Two hospitals processed residual positive BC samples from non-replicated patients using Molecular Mouse (MM) Sepsis panels (Alifax, Padova, Italy) for GP ID, GN ID and RD detection. Results were compared with an SOC workflow based on subculture, ID by MALDI-ToF mass spectrometry, phenotypic antibiogram, and real-time PCRs for RDs from isolated colonies. **Results:** A total of 140 and 136 residual positive BC samples were found to be valid for MM-ID and RD, respectively, yielding 76 GN and 76 GP species. Overall ID agreement at the species level was 136/152 (89%). RD agreement was 144/146 (99%). Regarding GN and GP species, ID agreement was 68/76 (89%) and 70/76 (92%), respectively. **Conclusions:** MM showed high sensitivity in RD detection; however, some discrepancies with results of the SoC workflow were observed, represented by reduced sensitivity for some species-specific IDs. Panel size and compact instrument dimension can be seen as the principal advantage of this modular molecular assay for the rapid detection of pathogens responsible for BSIs.

## 1. Introduction

Bloodstream infections (BSIs) are a major healthcare issue associated with high mortality and morbidity rates, particularly among immunocompromised and elderly patients [1]. The recent increase in antimicrobial resistance among Gram-negative (GN) and Gram-positive (GP) bacterial pathogens responsible for BSI has reduced the available therapeutic options, exacerbating the healthcare burden and worsening patients’ outcomes [2].

In this scenario, rapid diagnostic methods based on molecular testing for identification (ID) of pathogens and of relevant resistance determinants (RDs) from positive blood cultures (BCs) can provide valuable information for antimicrobial stewardship by allowing an earlier revision of empirical therapies in BSI management [3,4].

Several diagnostic solutions have recently been introduced for the rapid ID of pathogens from positive BCs, based on different methods ranging from Fluorescence In Situ Hybridization with Peptide Nucleic Acid Probes (PNA-FISH), to Multiplex Real-time Polymerase Chain Reaction (RT-PCR) combined with microarray-based assays, and whole genome sequencing (WGS). Some of these are already available on commercial diagnostic platforms, and are commonly used for their rapidity and reliability, even if they entail additional costs [5].

Molecular Mouse (MM, Alifax, Padova, Italy) is a molecular diagnostic system for positive BCs based on an RT-PCR approach, starting with the bacterial pellet recovered from the positive BC after a two-step centrifugation process. MM provides four cartridges, two for ID of Gram-negative (GN) pathogens and detection of relevant RDs, and two for ID of Gram-positive (GP) pathogens and detection of relevant RDs, that can be used in a modular fashion. Overall, MM targets a total of 30 bacterial species, four bacterial genera, one bacterial family, and 22 RDs for beta-lactams, glycopeptides and polymyxins [6,7,8] (Table S1), reporting results in 60 min (including preanalytical steps).

The aim of this study was to evaluate the performance of the MM system for the rapid ID of GN and GP pathogens and the detection of RDs from positive BCs, in comparison with a standard-of-care (SoC) workflow, with a case series from two different diagnostic laboratories operating in a setting of high resistance endemicity.

## 2. Materials and Methods

Residual positive BC samples (a vial from each case of positive BC, defining positivity as cases with at least one positive vial; or two different vials from two different sets for commensal organisms like coagulase-negative staphylococci) from non-replicated anonymized cases were collected from two hospitals in Tuscany (Careggi University Hospital, Florence, and San Giuseppe Hospital, Empoli). BCs had been processed with either the BACTEC™ FX (Becton Dickinson, Baltimore, MD, USA) or the BACT/ALERT^®^ VIRTUO^®^ (bioMérieux, Marcy l’Etoile, France) system. Each sample, after microscopy analysis of Gram-stained smears, underwent molecular analysis with the MM system, and SoC analysis by a conventional workflow.

The MM testing was performed according to the manufacturer’s instructions. Briefly, the positive BC was first centrifuged at 500× *g* for one minute to discard erythrocytes, and the supernatant was then centrifuged at 5000× *g* for one minute to collect the bacterial pellet. The latter was resuspended in 1 mL of sterile distilled water, and 5 µL was added to each well of the selected cartridge (Figure S1). Cartridge selection was based on the microscopy results after Gram-staining of the positive BC: when GP bacteria were detected, cartridges MM GRAM POS STAPH and/or GRAM POS NO STAPH; when GN bacteria were detected, cartridges MM GRAM NEG ID and MM GRAM NEG RES; when both GP and GN bacterial were detected, cartridges MM GRAM NEG ID, MM GRAM NEG RES, MM GRAM POS STAPH and GRAM POS NO STAPH (Table S1).

The conventional workflow consisted of subculturing the positive BC (10 µL) onto CPSE agar (bioMérieux). After incubation at 37 °C for 12–18 h, bacterial ID was performed with MALDI-ToF mass spectrometry (MALDI Biotyper^®^ Bruker, Franklin Lakes, NJ, USA, or Vitek^®^ MS, bioMérieux) from isolated colonies, and antimicrobial susceptibility testing (AST) was performed by a broth microdilution (BMD)-based system using lyophilized Micronaut plates (ITGN, ITGP and/or ITHMN, Merlin Diagnostika GmbH, Bornheim-Hersel, Germany) [9]. AST results were interpreted according to EUCAST v. 16.0 clinical breakpoints (available online: https://www.eucast.org/clinical_breakpoints (accessed on 29 January 2026)). Bacterial isolates were stored at −80 °C in Brain Hearth Infusion Broth (Liofilchem, Roseto degli Abruzzi, Italy) plus 30% *v*/*v* glycerol (Merck, Sigma-Aldrich, Rahway, NJ, USA). The presence or absence of RD as detected by MM was compared with results from AST and molecular analysis in bacterial isolates from the conventional workflow. For GN pathogens the molecular analysis covered *bla*_CTX-M_, *bla*_KPC_, *bla*_OXA-48_ (only for *Enterobacterales*), *bla*_NDM_, *bla*_VIM_, *bla*_IMP_ (for *Enterobacterales* and *Pseudomonas aeruginosa*), *bla*_OXA-23_ (only for *Acinetobacter baumannii*) and *mcr-1/2* genes (only for *Enterobacterales*) by RT-PCR, and *bla*_CMY-2-like_ genes (only for *Enterobacterales*) by an end-point PCR, as described previously [10,11]. Discrepancies between molecular results (including MM) and phenotype underwent further genotypic characterization by WGS. For GP pathogens the molecular analysis covered *vanA*/*vanB* (for enterococcal isolates) and *mecA/C* genes (for staphylococcal isolates) by RT-PCR, as described previously [12,13].

The MM results were compared with those from the SoC workflow for bacterial ID and detection of RD. In particular, full agreement was considered to have occurred when the MM results were fully consistent with the SoC results, including positive results for on-panel targets and negative results for off-panel targets. Disagreement was considered to have occurred when the MM results were negative for on-panel targets documented by the SoC workflow (false negatives, FNs) or were positive for on-panel targets not documented by the SoC workflow (false positives, FPs). For ID, agreement was also considered to have occurred in case of consistent results only at the genus or family level. With polymicrobial samples, comparative analysis was carried out for every isolate detected in the sample.

The comparative evaluation was analyzed in terms of percent diagnostic agreement (represented by the rate of agreement between MM and SoC at the species or genus/family level) between the MM results and those provided by the SoC workflow.

## 3. Results

**Figure 1:**
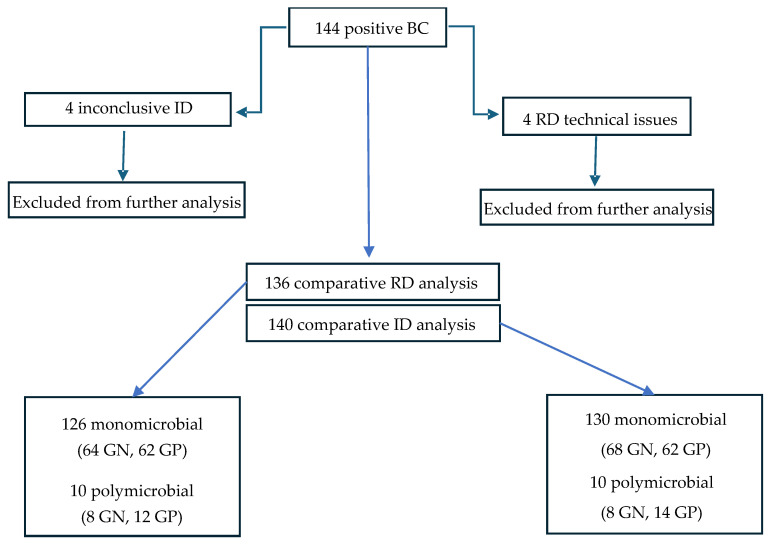
Flowchart of positive BC samples processed with MM. GN—Gram-negative; GP—Gram-positive; RD—resistance determinant; ID—species identification.

Of one hundred and forty-four positive BC samples processed with the MM system, four were excluded from the ID analysis because results were interpreted as inconclusive for ID (due to high C(t) values). Moreover, four samples were excluded from the RD analysis due to technical issues with the cartridges (failed run). Consequently, the comparative analysis was performed with 140 samples for ID and 136 samples for RD (Figure 1).

Processed by the SoC workflow, the 140 samples included in the comparative analysis for ID were 93% (130/140) monomicrobial and 7% (10/140) polymicrobial, yielding a total of 152 isolates (76 GN and 76 GP), while the 136 samples included in the comparative analysis for RD yielded a total of 146 isolates (72 GN and 74 GP) (Figure 1).

The 76 GN species considered for ID analysis included 63 *Enterobacterales* (*Klebsiella* spp., *n* = 29; *Escherichia coli*, *n* = 24; *Citrobacter* spp., *n* = 4; *Proteus mirabilis*, *n* = 2; and *Enterobacter cloacae* complex, *Morganella morganii*, *Serratia marcescens* and *Salmonella enterica*, *n* = 1 each) and 13 GN nonfermenters (*Acinetobacter baumannii* complex, *n* = 5; *Pseudomonas aeruginosa*, *n* = 3; *Stenotrophomonas maltophilia*, *n* = 4; and *Elizabethkingia miricola*, *n* = 1) (Table 1). The 76 GP species considered for ID analysis included *Staphylococcus aureus* (*n* = 16), coagulase-negative staphylococci (*n* = 26), *Enterococcus* spp. (*n* = 18), *Streptococcus* spp. (*n* = 13), *Bacillus* spp. (*n* = 2), and *Abiotrophia defectiva* (*n* = 1) (Table 2).

Regarding polymicrobial samples, two were positive for GN pathogens, five for GP, and three for both GN and GP (Table S2).

Regarding antimicrobial susceptibility by phenotypic characterization, 12% (8/63) of *Enterobacterales* were carbapenem-resistant, these being always associated with carbapenemase genes. A further 33% (22/63) were resistant to extended-spectrum cephalosporins, these always being correlated with CTX-M and/or CMY-2 production. Concerning Gram-negative nonfermenters, 100% (5/5) of *Acinetobacter baumannii* were carbapenem-resistant, and always associated with OXA-23 production, while 33% (1/3) of *Pseudomonas aeruginosa* exhibited a difficult-to-treat resistance phenotype. One isolate was negative for *bla*_VIM_, *bla*_NDM_ or *bla*_IMP_ carbapenemases, was subjected to WGS, and tested positive for the rare *bla*_FIM-1_ carbapenemase gene [14] (Table 3 and Table S3). Concerning GP, methicillin resistance was detected in 25% (4/16) of *S. aureus* isolates and in 77% (20/26) of CoNS, all being positive for these *mecA* gene, while 75% (6/8) of *E. faecium* isolates were vancomycin-resistant, all being positive for the *vanA* gene (Table 3 and Table S4).

### 3.1. Performance of Molecular Mouse in Species Identification

MM demonstrated an overall agreement of 136/152 (89%) at the species level, with fourteen false negatives and two false positives (Table 1 and Table 2). The overall diagnostic agreement (considering the species-level agreement and the false negatives recovered with genus/family targets) was 140/152 (92%).

Specifically, for GN species identification, MM showed an agreement of 68/76 (89%), achieving 100% agreement with most species except for *E. coli* (19/24, 79.1%), *Klebsiella aerogenes* (4/5, 80%) and *P. aeruginosa* (1/3, 33%) (Table 1). The genus/family level targets did not add any information on false negatives (Table 1). Only a single false positive was recorded (one *Enterobacteriaceae* spp., not confirmed by culture).

Concerning analysis of species identification for GP, MM showed an agreement of 70/76 (92%) at species level and 65/76 (85%) at genus level, with agreement of 100% for most targets except for *S. aureus* (15/16, 98%), *S. epidermidis* (81%, 9/11), *E. faecalis* (80%, 8/10) and *S. agalactiae* (0/1, 0%) (Table 2). The genus-level targets recovered information on two *S. epidermidis* and *two E. faecalis* (Table 2).

One false positive was registered for a sample which tested positive for both *Staphylococcus* spp. and *E. faecalis* MM targets, rather than for *E. faecalis* only, by culture.

The agreement for genus/family targets varied by group: 41/59 (69%) for *Enterobacteriaceae* spp., 0/2 (0%) for *Proteus* spp., 37/42 (88%) for *Staphylococcus* spp., 18/18 (100%) for *Enterococcus* spp., and 12/13 (92%) for *Streptococcus* spp. (Table 1 and Table 2).

Discrepancies observed in polymicrobial samples resulted in six false-negative findings: three samples involving GP, one involving GN, and one involving both. These missed identifications included *E. coli*, *S. agalactiae*, *S. epidermidis*, *Staphylococcus aureus* (detected only at the genus level) and *E. faecalis* (detected only at the genus level). Additionally, one false-positive result was recorded (*S. epidermidis*, in a sample containing only *K. pneumoniae* and *E. faecium*) (Table S2). Among discrepancies of polymicrobial samples 83% (5/6) involved GP species, in particular *S. epidermidis* (3/5).

### 3.2. Performance of Molecular Mouse in Identification of Resistance Determinants

MM RD performance, including false-positive and false-negative records, showed an overall agreement of 144/146 (99%). Regarding GN-related targets (*bla*_CMY-2_, *bla*_CTX-M_, *bla*_KPC_, *bla*_OXA-23_ and *mcr-1*) an overall agreement of 72/73 (99%) was registered, with only a FP detected (a sample positive for *bla*_CMY-2_ with MM and found negative with SOC molecular assay). Concerning GP-related targets (*mec*A and *van*A), agreement was 100% in all cases (Table 3).

## 4. Discussion

In recent years, the use of molecular syndromic platforms for diagnosis of bacteremia starting from positive BC has become increasingly popular in guiding antimicrobial stewardship interventions and in mitigating the impact of infections caused by multidrug-resistant pathogens. Rapid turnaround time and minimal hands-on time represent important advantages, in view of the introduction of these systems into diagnostic workflows for bloodstream infections [5].

This study evaluated the performance of MM, a modular molecular syndromic platform for the detection of bacterial pathogens and of clinically relevant RDs from positive BCs, under conditions representative of the diagnostic routine carried out in two different laboratories operating in an epidemiological setting affected by high endemicity of multidrug-resistant organisms. As such, it represents the first multicentric assessment of MM in comparison with a conventional SoC workflow.

The results of this evaluation showed that MM was able to rapidly identify the majority of bacterial pathogens grown in positive-BC vials, and of cognate RDs, in agreement with previous evaluations carried out with the MM system [15,16]. Some FNs (9%) were observed for pathogen ID, these being partially compensated for by genus- or family-specific targets, and the system showed excellent sensitivity for detection of on-panel RD. Moreover, the presence of genus- and family-specific targets allowed ID of some pathogens which are not covered by species-specific targets, expanding the diagnostic potential of the system. Almost half of the FNs for ID (6/14) were observed in cases of polymicrobial samples, suggesting that under these conditions the sensitivity could be reduced, in agreement with previous studies [16]. The two false positives (one for *Enterobacteriaceae* and one for *Staphylococcus* spp.) might be due to the presence of DNA from dead bacteria.

In the current scenario of molecular syndromic platforms for analysis of positive BC, the strengths of the MM system are represented by (i) the rapid turnaround time (60 min vs. the 30 to 150 min range of other methods) [17,18]; (ii) the coverage of some bacterial targets (e.g. *Streptococcus anginosus*, *Staphylococcus hominis*, *Staphylococcus haemolyticus*, *Staphylococcus sciuri*, *Staphylococcus saprophyticus*, *Staphylococcus xylosus*) and RD targets that may be missing in other platforms (e.g. OXA-23 and CMY-2) [19]; and (iii) the modular design, allowing for panel selection based on Gram-staining results, with potential for substantial economic savings, which could support the use of MM in smaller laboratories and in lower-income settings. Indeed, the cost of a single MM cartridge represents a ~45% cost reduction per sample, compared to other molecular platforms for the diagnosis of bloodstream infections. For GN bacteria identification, using both ID and RD cartridges remains less expensive overall. However, the MM system is limited by the lack of automation in the pre-analytical phase, as this requires two short centrifugation steps.

The limitations of this study included the absence of WGS data from isolated pathogens in cases of FN results of MM for bacterial ID, and the relatively low number of samples positives for rare MM targets.

In conclusion, the MM platform represents an interesting option among the available molecular syndromic panels for rapid analysis of positive BC samples, combining coverage of a broad array of bacterial pathogens and clinically relevant RDs with the possibility of modular use of different cartridges which may allow substantial economic advantages. This could be of notable value, particularly in challenging epidemiological scenarios, where multidrug-resistant pathogens are endemic. In addition, the rapid diagnosis of bloodstream infections is crucial for implementing effective targeted therapies that may improve clinical outcomes. Further studies, with larger number of samples, would be useful to confirm the potential of this diagnostic platform.

## Figures and Tables

**Table 1:** Agreement of ID results from MM for GN samples, in comparison with results from SOC. ^A^ Diagnostic agreement represents the rate of agreement between results from MM and SoC considering identification at the species or family level. ^B^ A total of 5 *E. coli* were not detected by both species and family probe (4 monomicrobial samples; 1 polymicrobial sample with *K. pneumoniae*) ^C^ 10 isolates of *K. pneumoniae* were not detected by family-specific probe (8 monomicrobial samples and 2 polymicrobial samples: one with *E. coli* and one with *E. faecium*). ^D^ One *K. aerogenes* from a monomicrobial sample tested negative with both species and family probes. ^E^ One *Klebsiella oxytoca* from a monomicrobial sample tested negative with family probe, including ^F^ 3 *Citrobacter koseri* (2/3 detected by family probe) and 1 *Citrobacter freundii* (detected by family probe). ^G^ A total of 2 *Proteus mirabilis* were detected by species probe but were not detected by genus-specific probe (1 monomicrobial sample 1 polymicrobial sample with *K. pneumoniae* and *E. faecalis*). ^H^ A total of 2 *P. aeruginosa* were not detected by species-specific probe (2 monomicrobial samples), including ^I^ 1 *Morganella morganii* and 1 *Elizabethkingia miricola*. NE—not evaluable.

Microorganism	No. of Isolates	Agreement with Species-Specific Probe (%)	Agreement with Family-Specific Probe (%)	Diagnostic Agreement (%) ^A^
***Escherichia coli***	24	19/24 (79) ^B^	19/24 (79) ^B^	19/24 (79)
***Klebsiella pneumoniae***	22	22/22 (100)	12/22 (54) ^C^	22/22 (100)
***Klebsiella aerogenes***	5	4/5 (80) ^D^	4/5 (80) ^D^	4/5 (80)
***Klebsiella oxytoca***	2	2/2 (100)	1/2 (50) ^E^	2/2 (100)
***Enterobacter cloacae*** **complex**	1	1/1 (100)	1/1 (100)	1/1 (100)
***Citrobacter*** **spp. ^F^**	4	NE	3/4 (75) ^F^	3/4 (75) ^F^
***Salmonella enterica***	1	1/1 (100)	1/1 (100)	1/1 (100)
***Proteus mirabilis*** **^G^**	2	2/2 (100)	-	2/2 (100)
***Serratia marcescens***	1	1/1 (100)	-	1/1 (100)
***Acinetobacter baumannii*** **complex**	5	5/5 (100)	-	5/5 (100)
***Pseudomonas aeruginosa***	3	1/3 (33) ^H^	-	1/3 (33)
***Stenotrophomonas maltophilia***	4	4/4 (100)	-	4/4 (100)
**GN off-panel ^I^**	2	NE	NE	NE

**Table 2:** Agreement of ID results for GP samples in comparison with results from SoC. ^A^ Diagnostic agreement represents the rate of agreement between results from MM and SoC, considering identification at the species or genus level. ^B^ One isolate of *S. aureus* was not detected by species-specific probe (polymicrobial sample with *E. coli*); two isolates were not detected by genus-specific probe (monomicrobial samples). ^C^ A total of 2 *S. epidermidis* were not detected by species-specific probe (1 monomicrobial sample and 1 polymicrobial with *S. hominis*). ^D^ A total of 2 *S. hominis* were undetected by genus-specific probe (1 monomicrobial sample and 1 polymicrobial with *E. faecalis*). ^E^ A total of 2 *E. faecalis* were undetected by species-specific probe (1 monomicrobial sample and 1 polymicrobial with *S. hominis*). ^F^ One *S. agalactiae* isolate from a polymicrobial sample (with *S. epidermidis*) was not detected by both species and genus-specific probes. ^G^ One *Streptococcus anginosus* was undetected by genus-specific probe. ^H^ One *Streptococcus agalactiae was* undetected by both species- and genus-specific probes. ^I^ One out of two *S. capitis* was not detected by genus-specific probe. ^J^ Off-panel *Streptococcus* spp. for species probe included 2 *S. oralis*, 2 *S. gallolyticus*, 1 *S. massiliensis* and 1 *S. sanguinis* (all detected by genus probe), ^K^ including 1 *S. parasanguinis*, 2 *S. mitis* 1 *Abiotrophia defective* and 2 *Bacillus* spp.; NE—not evaluable.

Microrganisms	No. of Isolates	Agreement with Species-Specific Probe (%)	Agreement with Genus-Specific Probe (%)	Overall Diagnostic Agreement (%) ^A^
***Staphylococcus aureus***	16	15/16 (98) ^B^	14/16 (87) ^B^	15/16 (98)
***Staphylococcus epidermidis***	11	9/11 (81) ^C^	11/11 (100)	11/11 (100)
***Staphylococcus hominis***	9	9/9 (100)	7/9 (77) ^D^	9/9 (100)
***Staphylococcus haemolyticus***	4	4/4 (100)	4/4 (100)	4/4 (100)
***Enterococcus faecalis***	10	8/10 (80) ^E^	10/10 (100)	10/10 (100)
***Enterococcus faecium***	8	8/8 (100)	8/8 (100)	8/8 (100)
***Streptococcus pneumoniae***	2	2/2 (100)	0/2 (0) ^F^	2/2 (100)
***Streptococcus anginosus***	1	1/1 (100)	0/1 (-) ^G^	1/1 (100)
***Streptococcus agalactiae***	1	0/1 (-) ^H^	0/1 (-) ^H^	0/1 (-)
**Other *Staphylococcus* spp. ^I^**	2	NE	1/2 (50)	1/2 (50)
**Other *Streptococcus* spp. ^J^**	6	NE	6/6 (100)	6/6 (100)
**Other GP off-panel ^K^**	6	NE	NE	NE

**Table 3:** Evaluation of agreement of MM test with RD targets.

	Resistance Mechanism	No. of Isolates	Agreement (%)
**GN**			
	*bla*_CMY-2_	3	3/3 (100)
	*bla*_CTX-M_	14	14/14 (100)
	*bla*_KPC_	1	1/1 (100)
	*bla*_CTX-M_ + *bla*_KPC_	7	7/7 (100)
	*bla*_OXA-23_	5	5/5 (100)
	*mcr-1*	1	1/1 (100)
	*None*	42	41/42 (98)
**Total GN**		73	72/73 (99)
**GP**	*mec*A	24	24/24 (100)
	*van*A	6	6/6 (100)
	*None*	43	43/43 (100)
**Total GP**		73	73/73 (100)
**Total GN + GP**		146	145/146 (99)

## Data Availability

The original contributions presented in this study are included in the article/Supplementary Material. Further inquiries can be directed to the corresponding author.

## Supplementary Materials

The following supporting information can be downloaded at: https://www.mdpi.com/article/10.3390/diagnostics16030481/s1, Figure S1. Schematic workflow of Molecular Mouse (MM) PBC pre-treatment steps. Table S1. Summary of the targets available on the Molecular Mouse system for each cartridge. Table S2. ID result of polymicrobial samples and agreement in comparison with SOC. Table S3. Antimicrobial susceptibility testing results for GN isolates. Table S4. Antimicrobial susceptibility testing results for GP isolates.
