# Supplementary material for: Evaluation of the Performance of Novel Gram-Negative and Gram-Positive Sepsis Panels for the Rapid Diagnosis of Bloodstream Infections
Source: Diagnostics (Basel). 2026 Feb 5;16(3):481. doi: 10.3390/diagnostics16030481 (PMC12897205; doi:10.3390/diagnostics16030481)
Supplement: Supplementary file 1 [file diagnostics-16-00481-s001.zip › diagnostics-4066108-supplementary.pdf]

Figure S1. Schematic workflow of Molecular Mouse (MM) PBC pre-treatment steps.

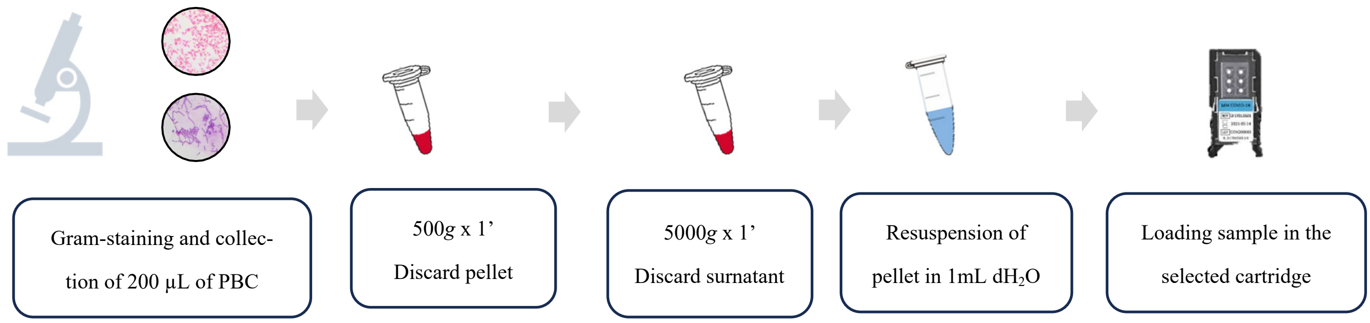

Table S1. Summary of the targets available on the Molecular Mouse system for each cartridge. GRAM NEG ID: target for identification of Gram-negative pathogens (species and genus targets), GRAM NEG RES: resistance determinants targets for Gram-negative pathogens; GRAM POS STAPH + RES: target for the identification of staphylococci and relative resistant determinants; GRAM POS NO STAPH + RES: target for identification of Gram-positive pathogens excluding staphylococci and relative resistant determinants.

MM GRAM NEG ID	MM GRAM NEG RES	MM GRAM POS STAPH + RES	MM GRAM POS NO STAPH + RES
Acinetobacter baumannii	bla_{KPC}	*Staphylococcus* spp.	*Bacillus subtilis*
Enterobacteriaceae	bla_{VIM}	*Staphylococcus aureus*	*Enterococcus* spp.
Klebsiella aerogenes	bla_{NDM}	*Staphylococcus epidermidis*	*Enterococcus faecalis*
Enterobacter cloacae	bla_{IMP}	*Staphylococcus haemolyticus*	*Enterococcus faecium*
Escherichia coli/Shigella spp.	$bla_{\mathrm{OXA\text{-}23\text{-}like}}$	*Staphylococcus lugdunensis*	*Listeria monocytogenes*
Haemophilus influenzae	$bla_{\mathrm{OXA\text{-}48\text{-}like}}$	*Staphylococcus sciuri*	*Streptococcus* spp.
Klebsiella oxytoca	bla_{SHV}	*Staphylococcus hominis*	*Streptococcus agalactiae*
Klebsiella pneumoniae	bla_{SHV} ESBL	*Staphylococcus simulans*	*Streptococcus anginosus*
Neisseria meningitidis	$bla_{\mathrm{CTX\text{-}M\text{-}1/9}}$ groups	*Staphylococcus saprophyticus*	*Streptococcus pneumoniae*
Proteus spp.	$bla_{\mathrm{CTX\text{-}M\text{-}2/8}}$ groups	*Staphylococcus xylosus*	*Streptococcus pyogenes*
Proteus mirabilis	$bla_{\mathrm{CMY\text{-}2}}$	*mecA*	*vanA*
Pseudomonas aeruginosa	*mcr-1*	*mecC*	*vanB*
Salmonella typhi	*mcr-2*	*SCCmec-orfX*	*vanC1*
Serratia marcescens		*vanA* and *vanB*	*vanC2/3*
Stenotrophomonas maltophilia			

Table S2. ID result of polymicrobial samples and agreement in comparison with SOC. Disagreement with species and genus/family-specific probes (missed ID of on-panel species) are highlighted in bold; species detected with MM and not in subculture (FP) are highlighted in red.

Polymicrobial ID (MALDI TOF)	MM ID (species and genus/family-specific probe)
Elizabethkingia miricola + *Stenotrophomonas maltophilia*	*Stenotrophomonas maltophilia* -
Klebsiella pneumoniae + ***Escherichia coli***	*Klebsiella pneumoniae*
Staphylococcus epidermidis + ***Streptococcus agalactiae***	*Staphylococcus epidermidis*
Staphylococcus hominis+ ***Staphylococcus epidermidis***	*Staphylococcus hominis*
Enterococcus faecalis+ *Staphylococcus hominis*	*Enterococcus* spp. + *Staphylococcus hominis*
Streptococcus mitis + *Streptococcus oralis*	*Streptococcus spp.*
Staphylococcus epidermidis + *Staphylococcus haemolyticus*	*Staphylococcus epidermidis* + *Staphylococcus haemolyticus*
Escherichia coli + ***Staphylococcus aureus***	*Escherichia coli* + *Staphylococcus* spp.
Proteus mirabilis + *Klebsiella pneumoniae* + ***Enterococcus faecalis*** + *Bacillus cereus*	*P. mirabilis* + *Klebsiella pneumoniae* + *Enterococcus faecalis*
Klebsiella pneumoniae + *Enterococcus faecium*	*Klebsiella pneumoniae* + *Enterococcus faecium* + *Staphylococcus epidermidis*

Table S3. Antimicrobial susceptibility testing results for GN isolates. *Including *Klebsiella pneumoniae* (n=22), *Klebsiella aerogenes* (n=5), *Klebsiella oxytoca* (n=2); $^{\Delta}$including *Citrobacter koseri* (n=1), *Citrobacter freundii* (n=3), *Proteus mirabilis* (n=2), *Morganella morganii* (n=1), *Enterobacter cloacae* (n=1), *Serratia marcescens* (n=1), *Salmonella enterica* (n=1); AMC: amoxicillin/clavulanic acid, CEP: cefepime, CAZ: ceftazidime, CAA: ceftazidime/avibactam, CTA: ceftolozane/tazobactam, CRO: ceftriaxone, ERT: ertapenem, MER: meropenem, PIT: piperacillin/tazobactam, COL: colistin.

GN						
**Strain**	***Klebsiella* spp.***	***Escherichia coli***	***Other Enterobacterales*** $^{\Delta}$	***Acinetobacter baumannii***	***Pseudomonas aeruginosa***	***Stenotrophomonas maltophilia***
**Number of isolates**	**29**	**24**	**10**	**5**	**3**	**4**
**AMC**	10/29 (34%)	19/24 (79%)	4/10 (40%)	-	-	-
**CEP**	12/29 (41%)	17/24 (71%)	6/10 (60%)	-	-	-
**CAZ**	8/29 (27%)	18/24 (75%)	5/10 (50%)	-	-	-
**CAA**	29/29 (100%)	24/24 (100 %)	10/10 (100%)	-	2/3 (66%)	-
**CTA**	20/29 (69%)	24/24 (100 %)	10/10 (100%)	-	2/3 (66%)	-
**CRO**	8/29 (27%)	17/24 (71%)	5/10 (50%)	-	-	-
**ERT**	21/29 (72%)	24/24 (100 %)	10/10 (100%)	-	-	-
**MER**	25/29 (86%)	24/24 (100 %)	10/10 (100%)	0	1/3 (33%)	-
**PIT**	13/29 (45%)	23/24 (96%)	10/10 (100%)	-	-	-
**COL**	29/29 (100%)	22/24 (92%)	4/10 (40%)	5/5 (100%)	2/3 (66%)	-

Table S4. Antimicrobial susceptibility testing results for GP isolates. §including *Staphylococcus epidermidis* (n= 11), *Staphylococcus haemolyticus* (n=4), *Staphylococcus capitis* (n=2), *Staphylococcus hominis* (n=9); £ including *Streptococcus sanguinis* (n=1), *Streptococcus parasanguinis* (n=1), *Streptococcus massilensis* (n=1), *Streptococcus mitis* (n=2), *Streptococcus anginosus* (n=1), *Streptococcus oralis* (n=2), *Streptococcus gallolyticus* spp. *pasteurianus* (n=2), *Streptococcus agalactiae* (n=1); OXA: oxacillin, TPL: teicoplanin, VAN: vancomycin.

GP						
**Strain**	***Staphylococcus aureus***	**CONs§**	***E. faecalis***	***E. faecium***	***Streptococcus pneumoniae***	***Viridans streptococci£***
**Number of isolates**	**16**	**26**	**10**	**8**	**2**	**11**
**OXA**	12/16 (75%)	6/26 (23%)	-	-	-	-
**TPL**	14/14 (100%)	24/26 (92%)	10/10 (100%)	2/8 (25%)	2/2 (100%)	11/11 (100%)
**VAN**	14/14 (100%)	26/26 (100%)	10/10 (100%)	2/8 (25%)	2/2 (100%)	11/11 (100%)
